# Supplementary material for: Structures of Mammeasins P and Q, Coumarin-Related Polysubstituted Benzofurans, from the Thai Medicinal Plant Mammea siamensis (Miq.) T. Anders.: Anti-Proliferative Activity of Coumarin Constituents against Human Prostate Carcinoma Cell Line LNCaP
Source: Pharmaceuticals (Basel). 2023 Feb 3;16(2):231. doi: 10.3390/ph16020231 (PMC9966789; doi:10.3390/ph16020231)
Supplement: Supplementary file 1 [file pharmaceuticals-16-00231-s001.zip › pharmaceuticals-2142896-supplementary.pdf]

Supplementary Materials

Structures of Mammeasins P and Q, Coumarin-Related Polysubstituted Benzofurans, from the Thai Medicinal Plant *Mammea siamensis* (Miq.) T. Anders.: Anti-Proliferative Activity of Coumarin Constituents against Human Prostate Carcinoma Cell Line LNCaP

Fenglin Luo [1], Yoshiaki Manse [1], Saowanee Chaipech [1,2], Yutana Pongpiriyadacha [3], Osamu Muraoka [1] and Toshio Morikawa [1,*]

[1] Pharmaceutical Research and Technology Institute, Kindai University, 3-4-1 Kowakae, Higashi-osaka 577-8502, Osaka, Japan
[2] Faculty of Agro-Industry, Rajamangala University of Technology Srivijaya, Thungyai, Nakhon Si Thammarat 80240, Thailand
[3] Faculty of Science and Technology, Rajamangala University of Technology Srivijaya, Thungyai, Nakhon Si Thammarat 80240, Thailand
* Correspondence: morikawa@kindai.ac.jp; Tel. +81-6-4307-4306; Fax: +81-6-6729-3577

Table of Contents

Figure S1. ^{1}H-NMR (500 MHz, $CDCl_3$) spectrum of mammeasin P (**1**) ···· S2
Figure S2. ^{13}C-NMR (125 MHz, $CDCl_3$) spectrum of mammeasin P (**1**) ···· S3
Figure S3. ^{1}H–^{1}H COSY spectrum of mammeasin P (**1**) ···· S4
Figure S4. HSQC spectrum of mammeasin P (**1**) ···· S5
Figure S5. HMBC spectrum of mammeasin P (**1**) ···· S6
Figure S6. ^{1}H-NMR (500 MHz, $CDCl_3$) spectrum of mammeasin Q (**2**) ···· S7
Figure S7. ^{13}C-NMR (125 MHz, $CDCl_3$) spectrum of mammeasin Q (**2**) ···· S8
Figure S8. ^{1}H–^{1}H COSY spectrum of mammeasin Q (**2**) ···· S9
Figure S9. HSQC spectrum of mammeasin Q (**2**) ···· S10
Figure S10. HMBC spectrum of mammeasin Q (**2**) ···· S11
Table S1. Anti-proliferative effects of coumarin constituents (**3**–**20**, **24**–**28**, **30**, **33**–**38**, **40**–**47**) from *M. siamensis* flowers against LNCaP cells ···· S12
Table S2. IC_{50} values of coumarin constituents (**3**–**12**, **15**, **16**, **18**–**21**, **24**–**38**, **40**–**47**) from *M. siamensis* flowers against testosterone 5α-reductase ···· S14

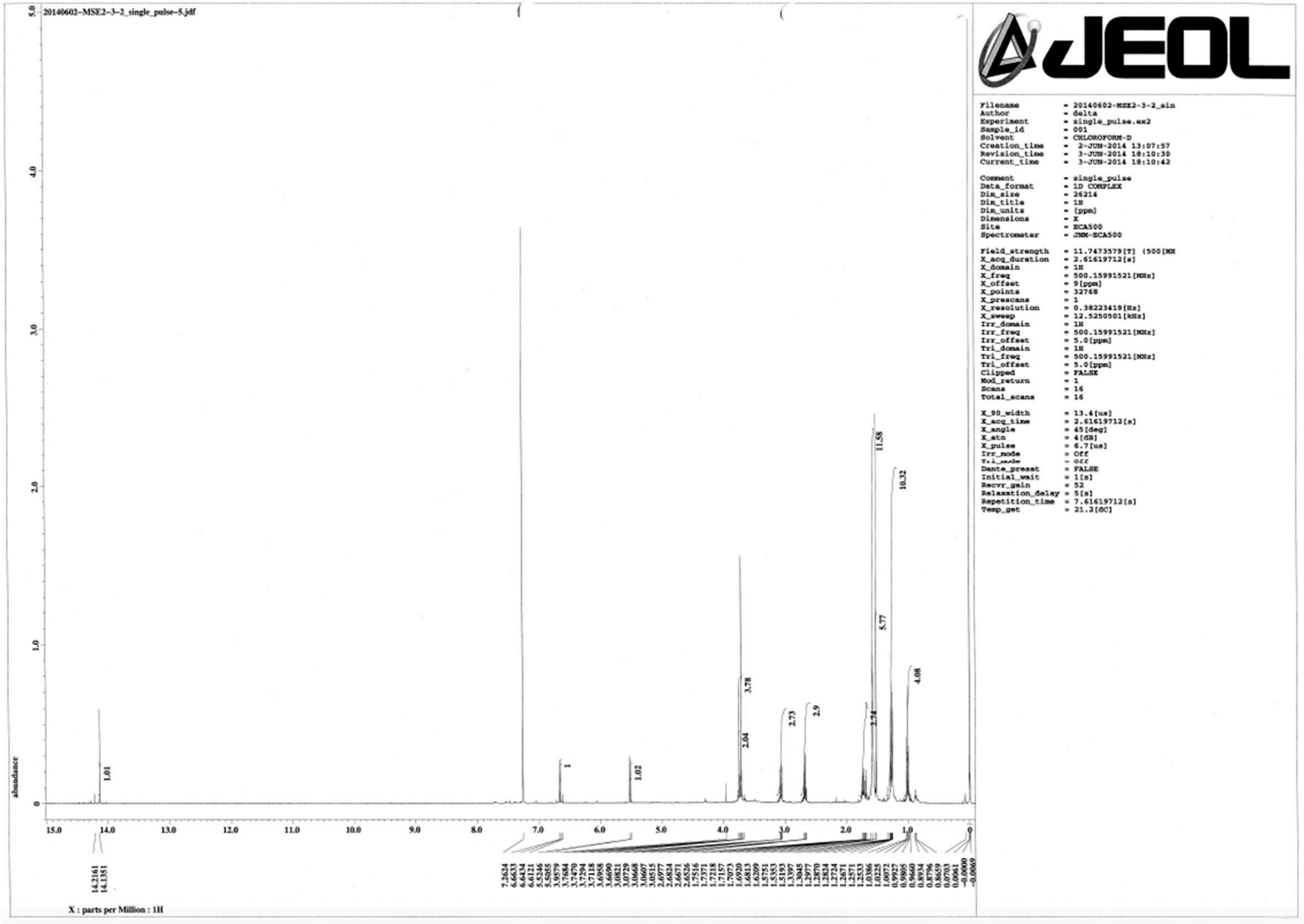

Figure S1. ^{1}H-NMR (500 MHz, $CDCl_3$) spectrum of mammeasin P (**1**).

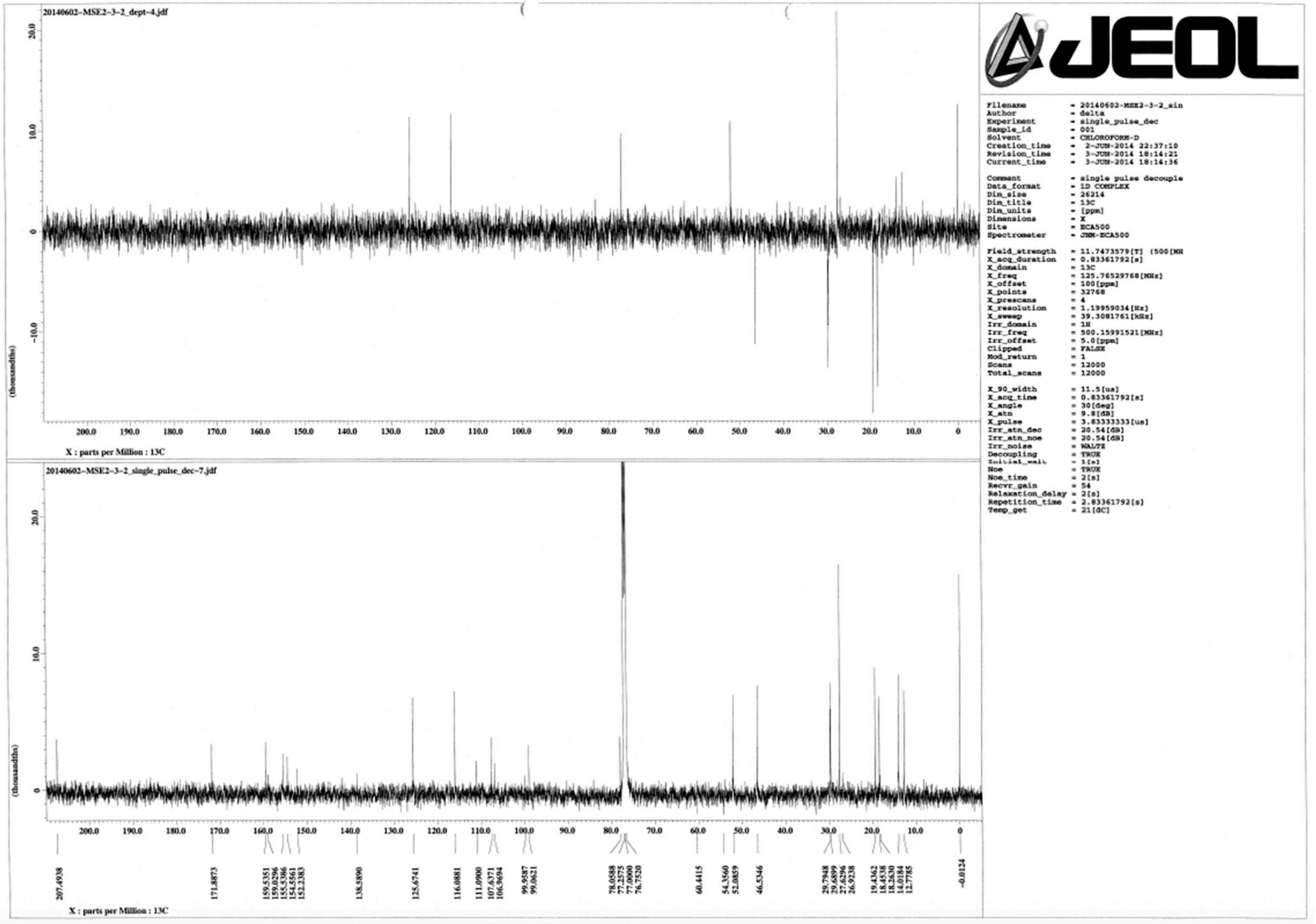

Figure S2. ^{13}C-NMR (125 MHz, $CDCl_3$) spectrum of mammeasin P (**1**).

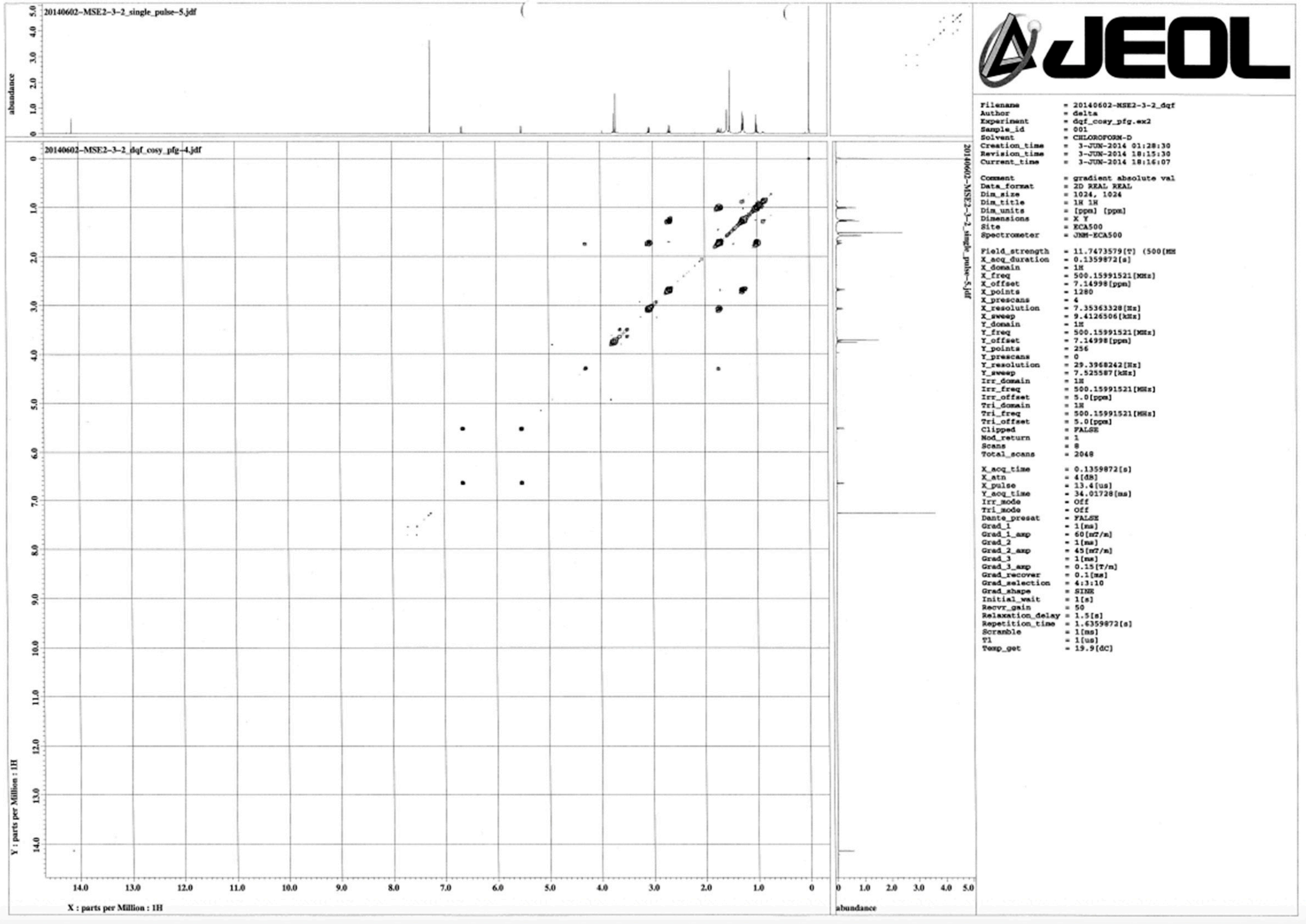

Figure S3. ^1H–^1H COSY spectrum of mammeasin P (**1**).

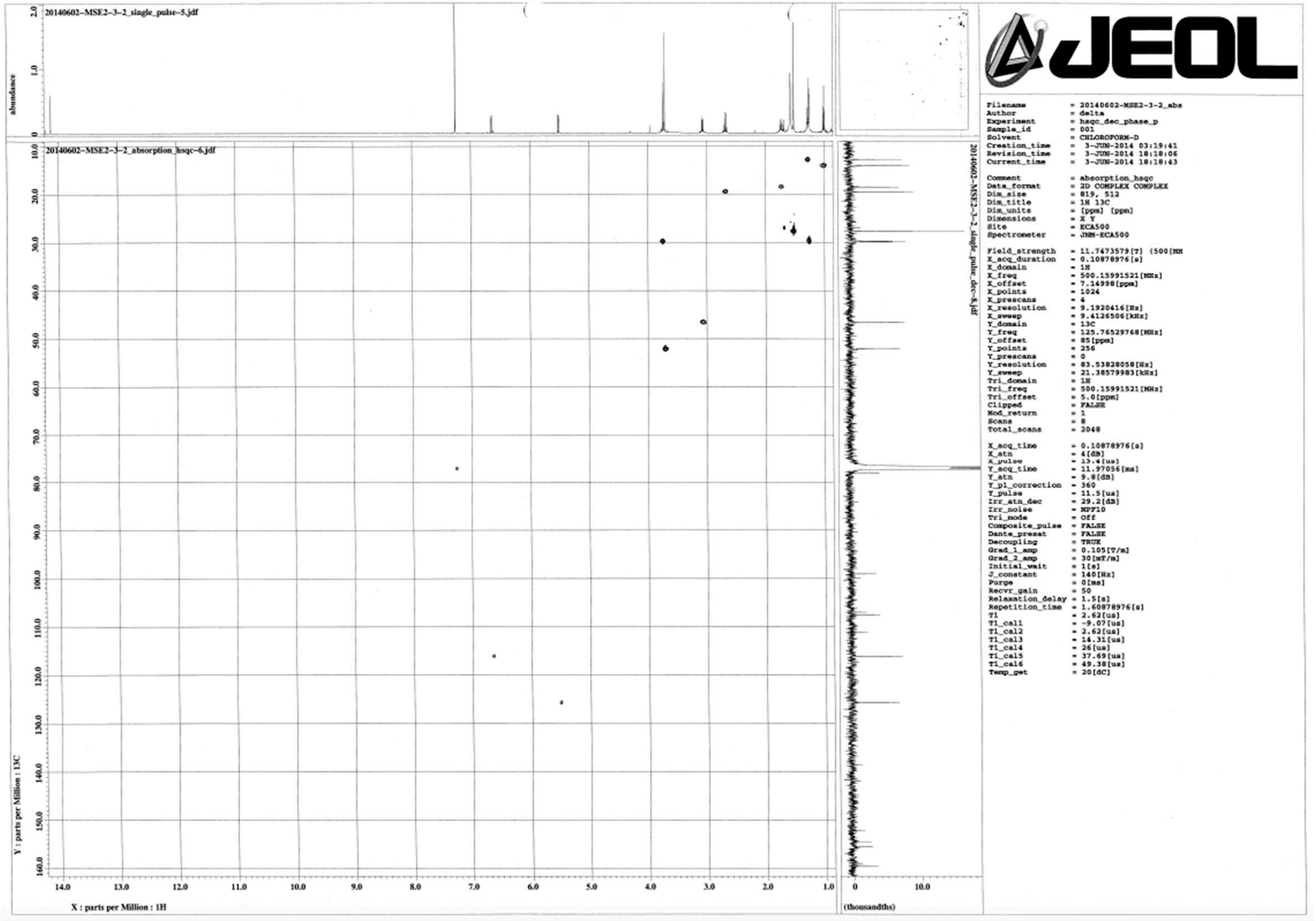

Figure S4. HSQC spectrum of mammeasin P (**1**).

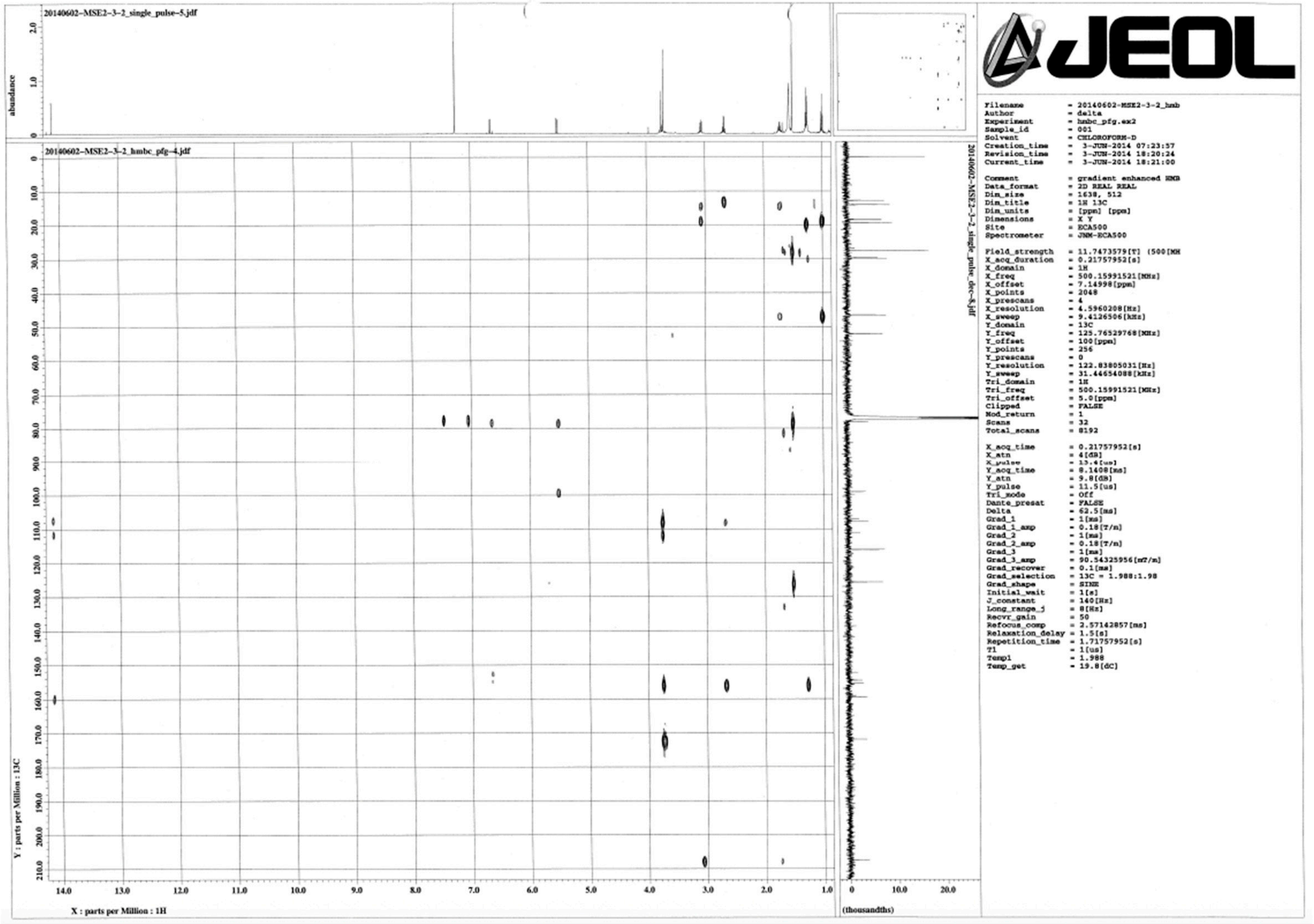

Figure S5. HMBC spectrum of mammeasin P (**1**).

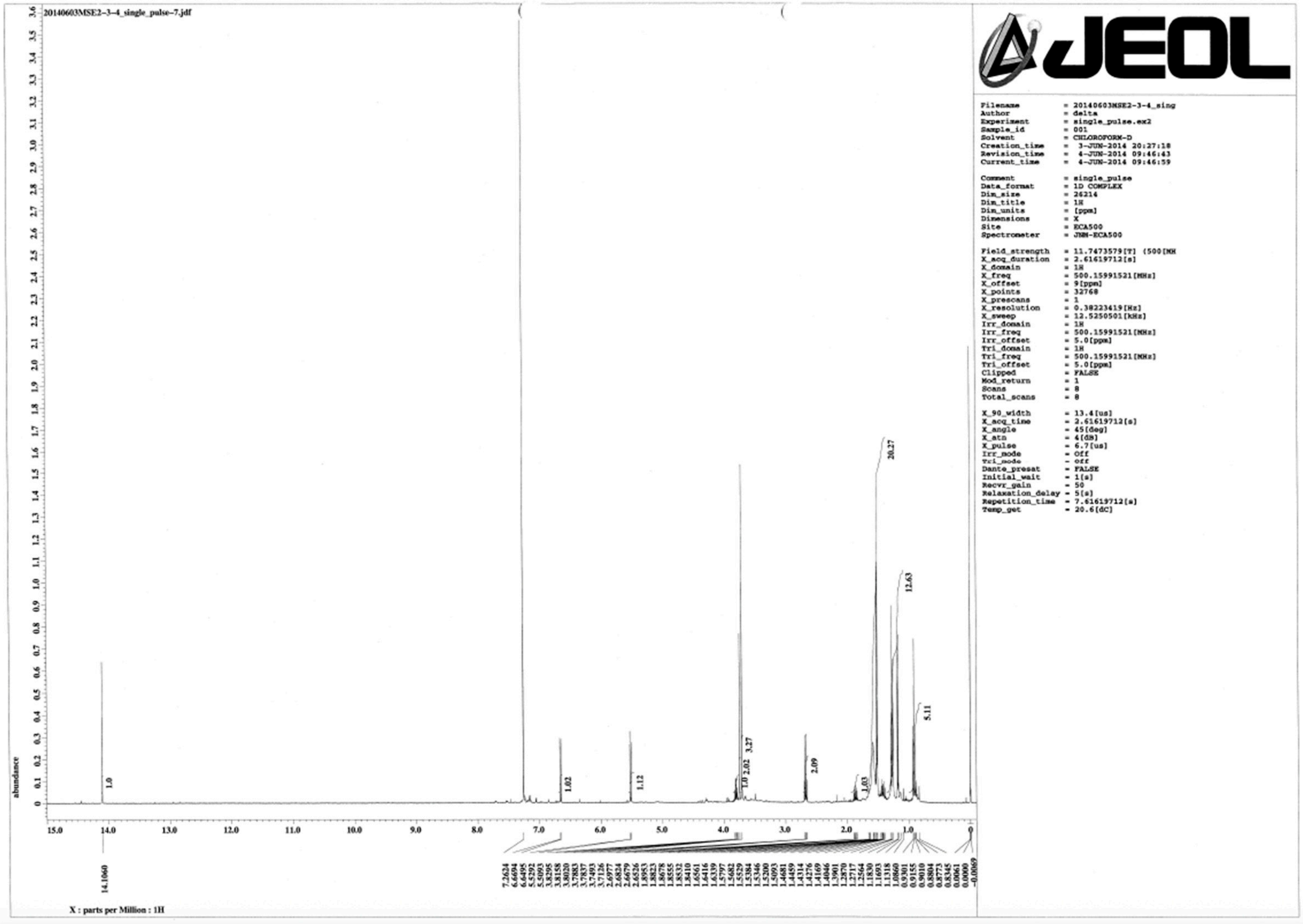

Figure S6. ^{1}H-NMR (500 MHz, $CDCl_3$) spectrum of mammeasin Q (**2**).

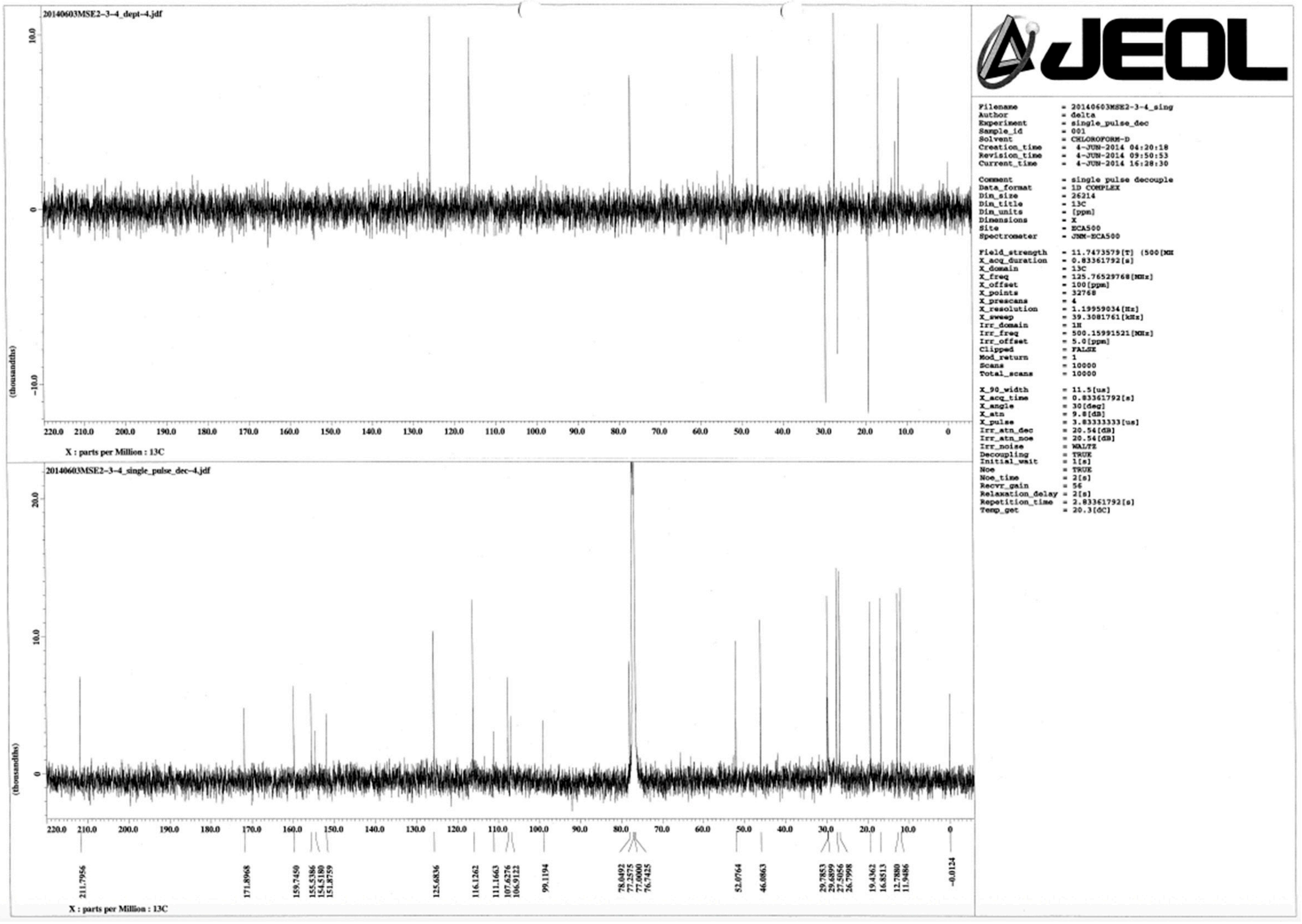

Figure S7. ^{13}C-NMR (125 MHz, $CDCl_3$) spectrum of mammeasin Q (**2**).

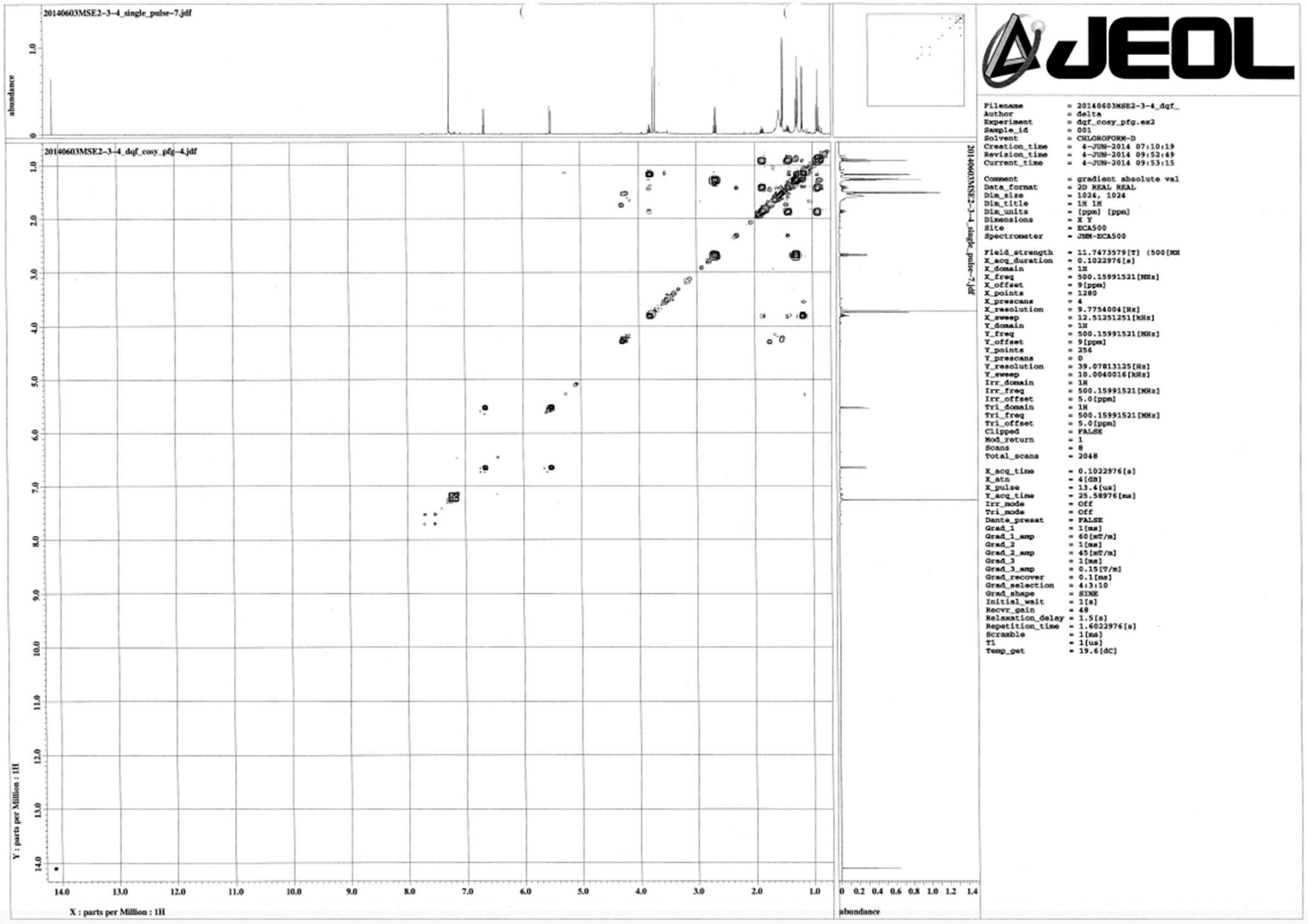

Figure S8. ^1H–^1H COSY spectrum of mammeasin Q (**2**).

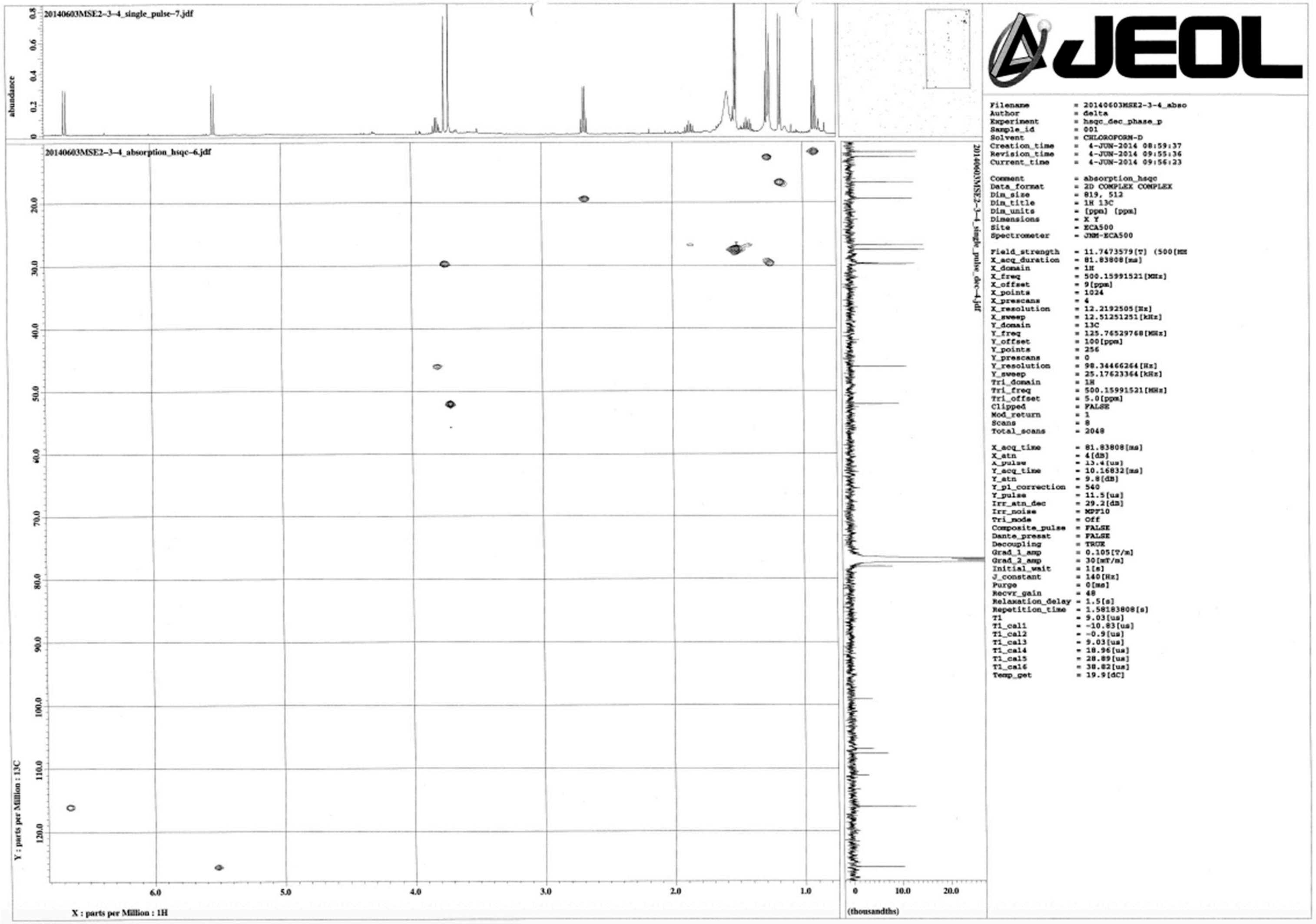

Figure S9. HSQC spectrum of mammeasin Q (**2**).

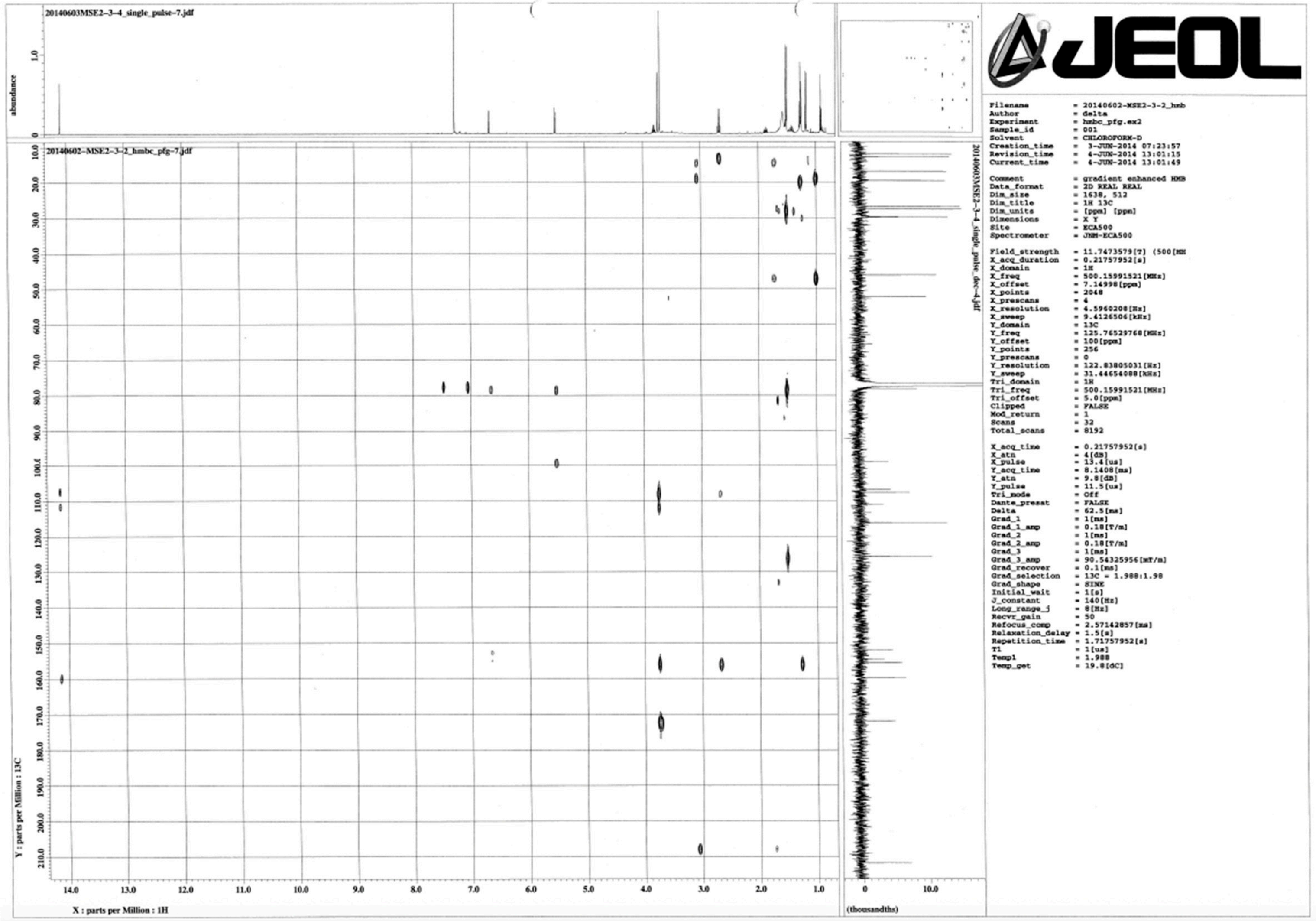

Figure S10. HMBC spectrum of mammeasin L (**2**)

Table S1. Anti-proliferative effects of coumarin constituents (**3–20**, **24–28**, **30**, **33–38**, **40–47**) from *M. siamensis* flowers against LNCaP cells.

**Treatment**	**Inhibition (%)**					IC50 (μM)
	**0 μM**	**0.1 μM**	**0.3 μM**	**1 μM**	**3 μM**	
Mammeasin A (**3**)	100.0 ± 1.1	95.3 ± 2.9	97.4 ± 1.9	60.0 ± 2.6**	12.9 ± 0.1**	1.2
Mammeasin B (**4**)	100.0 ± 1.3	93.1 ± 3.2	86.3 ± 2.1**	28.0 ± 1.7**	13.2 ± 0.3**	0.63
Surangin B (**18**)	100.0 ± 2.3	94.8 ± 1.7	94.1 ± 2.2	88.7 ± 0.6**	15.3 ± 0.3**	1.5
Mammea E/BA (**40**)	100.0 ± 2.9	113.1 ± 5.5	109.4 ± 4.8	51.6 ± 1.6**	12.6 ± 0.4**	0.88
Mammea E/BB (**41**)	100.0 ± 1.9	92.1 ± 1.9*	82.8 ± 2.7**	16.1 ± 0.7**	9.6 ± 0.1**	0.52
Mammea E/BC (**42**)	100.0 ± 2.1	88.5 ± 1.5**	30.2 ± 1.1**	11.1 ± 0.1**	8.6 ± 0.1**	0.12
	**0 μM**	**0.3 μM**	**1 μM**	**3 μM**	**10 μM**	
Kayeassamin E (**24**)	100.0 ± 1.1	99.5 ± 2.1	91.0 ± 2.3**	51.2 ± 1.1**	9.2 ± 0.1**	3.0
Kayeassamin G (**26**)	100.0 ± 0.7	96.5 ± 2.7	93.4 ± 3.9	60.0 ± 2.7**	8.9 ± 0.2**	3.5
	**0 μM**	**30 μM**	**50 μM**	**60 μM**	**100 μM**	
Mammea E/BD cyclo D (**44**)	100.0 ± 2.1	92.4 ± 4.7	69.9 ± 4.2**	36.2 ± 1.1**	18.8 ± 0.7**	53.9
	**0 μM**	**3 μM**	**10 μM**	**30 μM**	**100 μM**	
Mammeasin C (**5**)	100.0 ± 1.7	111.7 ± 3.4*	96.5 ± 3.5	54.1 ± 2.2**	8.4 ± 0.1**	30.5
Mammeasin D (**6**)	100.0 ± 3.1	92.1 ± 3.1	76.0 ± 3.0**	58.1 ± 3.2**	8.0 ± 0.1**	25.0
Mammeasin E (**7**)	100.0 ± 2.7	94.8 ± 3.9	24.8 ± 1.4**	8.9 ± 0.0**	8.2 ± 0.3**	5.9
Mammeasin F (**8**)	100.0 ± 1.6	90.4 ± 4.8*	79.8 ± 2.3**	15.5 ± 0.5**	7.3 ± 0.0**	16.7
Mammeasin G (**9**)	100.0 ± 4.3	115.2 ± 6.9	102.6 ± 3.6	80.6 ± 2.2*	40.6 ± 2.4**	83.5
Mammeasin H (**10**)	100.0 ± 2.1	97.1 ± 1.4	95.4 ± 1.3	90.8 ± 1.2*	31.5 ± 1.1**	69.4
Mammeasin I (**11**)	100.0 ± 2.7	107.9 ± 1.4	110.1 ± 5.0	100.7 ± 2.7	47.9 ± 0.8**	*ca* 100
Mammeasin J (**12**)	100.0 ± 3.4	97.1 ± 2.9	99.1 ± 1.8	91.4 ± 2.4	86.9 ± 3.8*	>100
Mammeasin K (**13**)	100.0 ± 1.7	95.8 ± 2.3	95.6 ± 1.5	94.4 ± 1.8	79.9 ± 1.6**	>100
Mammeasin L (**14**)	100.0 ± 1.8	92.7 ± 1.6*	88.9 ± 2.2**	83.9 ± 1.7**	19.6 ± 0.6**	49.4
Mammeasin M (**15**)	100.0 ± 6.2	95.0 ± 4.8	95.5 ± 2.2	87.0 ± 5.5	91.3 ± 0.8	>100
Mammeasin N (**16**)	100.0 ± 1.6	97.2 ± 4.6	92.3 ± 2.2	80.3 ± 3.1**	49.4 ± 1.7**	*ca* 100
Mammeasin O (**17**)	100.0 ± 1.1	93.2 ± 2.3	91.0 ± 4.4*	61.3 ± 1.4**	15.1 ± 0.3**	35.2
Surangin C (**19**)	100.0 ± 4.2	105.3 ± 7.4	54.4 ± 4.7**	14.2 ± 1.0**	7.9 ± 0.0**	11.8
Surangin D (**20**)	100.0 ± 2.2	110.4 ± 4.2*	79.9 ± 3.3**	20.4 ± 0.4**	7.5 ± 0.1**	24.7
Kayeassamin F (**25**)	100.0 ± 2.8	78.8 ± 1.0**	21.9 ± 0.7**	8.6 ± 0.2**	7.4 ± 0.1**	6.2
Mammea A/AA (**28**)	100.0 ± 1.6	97.6 ± 2.9	98.8 ± 0.5	87.9 ± 2.0**	17.9 ± 0.5**	51.9
Mammea A/AC (**30**)	100.0 ± 1.2	105.2 ± 2.6	75.9 ± 3.6**	13.8 ± 0.3**	15.1 ± 0.3**	26.2
Mammea A/AB cyclo D (**33**)	100.0 ± 3.0	90.8 ± 3.0*	91.5 ± 2.9	87.7 ± 1.8**	82.7 ± 0.4**	>100

Mammea A/AC cyclo D (**34**)	100.0 ± 0.8	95.2 ± 1.2	95.7 ± 1.9	92.6 ± 2.5*	90.0 ± 1.0**	>100
Mammea A/AA cyclo F (**35**)	100.0 ± 1.4	94.8 ± 3.4	77.6 ± 2.2**	34.9 ± 1.8**	6.6 ± 0.1**	21.3
Mammea A/AC cyclo F (**36**)	100.0 ± 2.8	88.2 ± 4.2*	69.5 ± 2.9**	44.1 ± 1.2**	14.1 ± 0.2**	39.7
Mammea B/AB cyclo D (**37**)	100.0 ± 2.9	102.7 ± 3.5	97.6 ± 2.7	87.0 ± 3.4*	28.3 ± 0.6**	61.9
Mammea B/AC cyclo D (**38**)	100.0 ± 1.7	86.9 ± 1.5**	85.8 ± 4.3**	82.6 ± 1.1**	78.4 ± 1.4**	>100
Mammea E/BC cyclo D (**43**)	100.0 ± 2.0	99.8 ± 3.2	94.2 ± 4.7	34.8 ± 1.2**	7.6 ± 0.0**	23.1
Deacetylmammea E/AA cyclo D (**45**)	100.0 ± 1.3	90.8 ± 3.5**	85.0 ± 0.4**	39.3 ± 0.7**	6.7 ± 0.1**	25.9
Deacetylmammea E/BB cyclo D (**46**)	100.0 ± 1.0	91.8 ± 1.9*	86.0 ± 3.0**	51.7 ± 1.6**	7.0 ± 0.0**	34.0
Deacetylmammea E/BC cyclo D (**47**)	100.0 ± 2.0	94.5 ± 1.3*	85.7 ± 0.9**	24.7 ± 0.7**	6.9 ± 0.0**	19.7
	**0 μM**	**10 μM**	**20 μM**	**30 μM**	**50 μM**	
Kayeassamin I (**27**)	100.0 ± 1.7	79.8 ± 2.1**	36.4 ± 1.1**	16.6 ± 0.6**	6.5 ± 0.1**	16.1
	**0 nM**	**0.3 μM**	**1 nM**	**3 nM**	**10 nM**	**IC_{50} (nM)**
Paclitaxel [41,42]	100.0±4.0	85.5±2.8*	73.4±3.0**	57.1±4.3**	16.8±1.0**	3.7

Each value represents the mean ± standard error of the mean (S.E.M.) (*N* = 5). Significantly different from the control, * p <0.05, ** p <0.01 (Dunnett).

Table S2. IC_{50} values of coumarin constituents (**3–12**, **15**, **16**, **18–21**, **24–38**, **40–47**) from *M. siamensis* flowers against testosterone 5α-reductase.

Treatment	IC_{50} (μM)	Treatment	IC_{50} (μM)
Mammeasin A (**3**)[*)]	19.0	Mammea A/AA (**28**)[*)]	19.5
Mammeasin B (**4**)[*)]	24.0	Mammea A/AB (**29**)[*)]	>100 (23.3)[a)]
Mammeasin C (**5**)[*)]	91.9	Mammea A/AC (**30**)[*)]	>100 (41.5)[a)]
Mammeasin D (**6**)[*)]	>100 (16.4)[a)]	Mammea A/AD (**31**)[*)]	>100 (30.3)[a)]
Mammeasin E (**7**)[*)]	22.6	Mammea A/AA cyclo D (**32**)[*)]	>100 (38.3)[a)]
Mammeasin F (**8**)[*)]	>100 (14.9)[a)]	Mammea A/AB cyclo D (**33**)[*)]	>100 (6.7)[a)]
Mammeasin G (**9**)	>100 (31.3)[a)]	Mammea A/AC cyclo D (**34**)[*)]	>100 (32.0)[a)]
Mammeasin H (**10**)	>100 (37.2)[a)]	Mammea A/AA cyclo F (**35**)[*)]	23.6
Mammeasin I (**11**)	>100 (29.6)[a)]	Mammea A/AC cyclo F (**36**)[*)]	83.8
Mammeasin J (**12**)	>100 (31.5)[a)]	Mammea B/AB cyclo D (**37**)[*)]	>100 (40.7)[a)]
Mammeasin M (**15**)	>100 (18.8)[a)]	Mammea B/AC cyclo D (**38**)[*)]	>100 (27.3)[a)]
Mammeasin N (**16**)	>100 (39.6)[a)]	Mammea E/BA (**40**)	16.2
Surangin B (**18**)[*)]	>100 (38.5)[a)]	Mammea E/BB (**41**)[*)]	16.8
Surangin C (**19**)[*)]	5.9	Mammea E/BC (**42**)[*)]	>100 (19.1)[a)]
Surangin D (**20**)[*)]	19.5	Mammea E/BC cyclo D (**43**)[*)]	>100 (31.9)[a)]
Kayeassamin A (**21**)[*)]	>100 (20.2)[a)]	Mammea E/BD cyclo D (**44**)	>100 (31.7)[a)]
Kayeassamin E (**24**)[*)]	33.8	Deacetylmammea E/AA cyclo D (**45**)[*)]	>100 (37.1)[a)]
Kayeassamin F (**25**)[*)]	15.9	Deacetylmammea E/BB cyclo D (**46**)[*)]	>100 (31.9)[a)]
Kayeassamin G (**26**)[*)]	17.7	Deacetylmammea E/BC cyclo D (**47**)[*)]	>100 (40.8)[a)]
Kayeassamin I (**27**)[*)]	>100 (37.6)[a)]	Finasteride[*)]	0.12

Each value represents the mean ± S.E.M. (*N* = 3–4). [a)]Values in parentheses represent inhibition % at 100 μM. [*)]Ref. [28]

Assay for Testosterone 5α-Reductase Inhibitory Activity: The experiment was performed in accordance with previously reported methods [28]. In brief, the assay was performed in 48-well microplates (Sumitomo Bakelite Co., Ltd., Tokyo, Japan). The reaction solution was pre-incubated with or without a test sample (5 μL/well, dissolved in DMSO), in a potassium phosphate buffer (40 mM, pH 6.5, 490 μL/well) containing substrate (0.35 nmol of testosterone, Tokyo Chemical Industry Co., Ltd., Tokyo, Japan) and NADPH (10 nmol, Oriental Yeast Co., Ltd., Tokyo, Japan) at room temperature (25°C) for 20 min. The enzymatic reaction was initiated by the addition of rat liver S9 fractions (10 μL/well, dissolved in the phosphate buffer, 20.6 μg/well, Oriental Yeast Co., Ltd., Tokyo, Japan, lot no. 109031513) at 37 °C for 30 min. After incubation, the reaction mixture was immediately heated in boiling water for 2 min to stop the reaction. Then the reaction solution of each well was transferred to a microtube and extracted with 500 μL of EtOAc. After the microtube was centrifuged (10,000 rpm, 5 min), an aliquot of each EtOAc phase (300 μL) was transferred into another tube. The solvent in the tube was evaporated and the residue was dissolved in 30 μL of acetonitrile containing an internal standard (I.S.) fludrocortisone acetate (20 μg/mL, Sigma-Aldrich, Co., LLC, St. Louis, USA). An aliquot of 2 μL was injected into the HPLC under the following conditions [Instrument: a series LC-20A Prominence HPLC system (Shimazdu Co., Kyoto, Japan); Detection: UV (254 nm); Column: Cosmosil $5C_{18}$-MS-II (Nakalai Tesque Inc., Kyoto, Japan, 5 μm particle size, 2.0 mm i.d. × 150 mm); Column temperature: 40°C; Mobile phase: MeOH–H_2O (60:40, v/v); Flow rate: 0.2 mL/min; retention time: 13.5 min for testosterone and 8.0 min for I.S. A similar procedure that described above was carried out for the control tubes. The 5α-reductase inhibitory activity was determined from the following equation using the peak area ratios (*r* = testosterone / I.S.). Experiments were performed in triplicate or quadruple, and IC_{50} values were determined graphically. The 5α-reductase inhibitor finasteride (Tokyo Chemical Industry Co., Ltd., Tokyo, Japan) was used as a reference compound.

$$\text{Inhibition (\%)} = [r\,(\mathrm{T}) - r\,(\mathrm{C}) \,/\, r\,(\mathrm{B}) - r\,(\mathrm{C})] \times 100$$

Control (C): enzyme (+), test sample (–); Test (T): enzyme (+), test sample (+); Blank (B): enzyme (–), test sample (+)
